# PITX1 is a reliable biomarker for predicting prognosis in patients with oral epithelial dysplasia

**DOI:** 10.3892/ol.2013.1775

**Published:** 2013-12-24

**Authors:** MOTOKI NAKABAYASHI, MITSUHIKO OSAKI, ISAMU KODANI, FUTOSHI OKADA, KAZUO RYOKE, MITSUO OSHIMURA, HISAO ITO, HIROYUKI KUGOH

**Affiliations:** 1 Division of Organ Pathology, Department of Microbiology and Pathology, Tottori University Faculty of Medicine, Tottori University Graduate School of Medical Science, Yonago, Tottori 683-8503, Japan; 2 Division of Oral and Maxillofacial Biopathological Surgery, Department of Sensory and Motor Organ Medicine, Tottori University Faculty of Medicine, Tottori University Graduate School of Medical Science, Yonago, Tottori 683-8503, Japan; 3 Department of Oral and Maxillofacial Surgery, Matsue Red Cross Hospital, Matsue, Shimane 690-8506, Japan; 4 Division of Pathological Biochemistry, Department of Biomedical Sciences, Tottori University Faculty of Medicine, Yonago, Tottori 683-8503, Japan; 5 Division of Molecular Genetics and Biofunction, Institute of Regenerative Medicine and Biofunction, Tottori University Graduate School of Medical Science, Yonago, Tottori 683-8503, Japan

**Keywords:** PITX1, oral epithelial dysplasia, immunohistochemistry, biomarker

## Abstract

Paired-like homeodomain 1 (PITX1) genes are essential in human development. In the present study, PITX1 protein expression was evaluated in human normal oral mucosa, oral epithelial dysplasia and oral squamous cell carcinoma (OSCC), with the aim of examining the expression patterns of these critical genes during the multi-stage transformation of oral epithelial dysplasia to OSCC. PITX1 and Ki-67 expression were assessed by immunohistochemistry in 26 individuals with normal oral mucosa, 106 patients with oral epithelial dysplasia and 97 OSCC patients. The labeling indices (LIs) of PITX1 and Ki-67 were calculated and their correlation with the incidence of malignancy was evaluated. The PITX1 LI of the dysplasia specimens was significantly lower than that of the normal oral mucosa samples, but significantly higher than that of the OSCC samples. The oral epithelial dysplasia patients that exhibited low PITX1 expression showed a significantly higher incidence of malignant transformation than those exhibiting high PITX1 expression, regardless of the histological grades of their oral epithelial dysplasias. On the other hand, no correlation was observed between the Ki-67 LI and the incidence of malignancy. These results suggested that PITX1 suppression is associated with malignant transformation in the oral epithelium and that PITX1 expression may serve as a novel biomarker for predicting prognosis in oral epithelial dysplasia.

## Introduction

Oral leukoplakia is the most common type of premalignant lesion affecting the oral mucosa (1,2). Occasionally, types of oral cancer are preceded by clinically evident potential malignant oral disorders. Leukoplakia is the most common of these disorders and exhibits a malignant transformation rate ranging between 0.6 and 18% (3). Its malignant transformation rate may be directly associated with the severity of epithelial dysplasia, as it ranges between 5% for leukoplakia with mild epithelial dysplasia and 43% for leukoplakia with severe epithelial dysplasia (4). Thus, the risk of the malignant transformation of leukoplakia may be evaluated based on microscopic assessments of epithelial dysplasia (5–7). Oral squamous cell carcinoma (OSCC) is considered to develop from precancerous dysplastic lesions via multi-step carcinogenic processes, in which oncogene activation and the loss of tumor suppressor gene expression are the key features (8).

As a family of transcription factors, homeobox genes are not only important in embryonic development and differentiation, but also control the differentiation and proliferation of mature tissues (9). Paired-like homeodomain 1 (PITX1) was originally described as a bicoid-related homeobox transcription factor that is involved in the transcription of the proopiomelanocortin gene in the adult pituitary. In addition, PITX1 is involved in the differentiation of pituitary cells and the formation of the pituitary gland (10). PITX1 is exclusively expressed throughout the developing hindlimb, but not the forelimb bud; it determines the morphology of the muscles, tendons and bones of the hindlimbs (11,12). The development of the oral epithelium, the first branchial arch and its derivatives, are also known to require PITX1 (13,14).

In previous studies, the downregulation of PITX1 expression has been consistently associated with human OSCC (15), esophageal (16), gastric (17,18), bronchial (19,20), hepatic (21), colorectal (22), pancreatic (23) and prostatic (24) malignancies, as well as malignant melanoma (18,25). However, the clinical significance of PITX1 expression in the development of OSCC remains unclear. In the present study, PITX1 expression was examined in oral epithelial dysplasia, which is considered to be a precancerous lesion of OSCC.

## Materials and methods

### Tissue samples

Tissue samples were analyzed from 26 individuals with normal oral mucosa, 106 cases of oral epithelial dysplasia and 97 OSCC patients. All the specimens were obtained from the Division of Oral and Maxillofacial Biopathological Surgery, Tottori University Faculty of Medicine (Yanago, Japan). Approval for the study was obtained from the institutional review board of Tottori University Faculty of Medicine (no. 1558). All specimens were fixed in 10% buffered formalin and embedded in paraffin wax. The resultant paraffin blocks were sectioned into 4-μm slices. All patients provided written informed consent.

The histological diagnosis of OSCC and oral epithelial dysplasia was performed according to the World Health Organization criteria for the histological typing of cancer and precancer of the oral mucosa (26).

### Immunohistochemistry

All the specimens were fixed with 10% formalin and embedded in paraffin. Histofine SAB-PO^®^ immunohistochemical staining kit (Nichirei Corporation, Tokyo, Japan) and 4-μm-thick sections were used for the immunohistochemical analysis. As primary antibodies, a rabbit polyclonal antibody raised against PITX1 (1:800; Abcam, Cambridge, UK) and a mouse monoclonal antibody raised against Ki-67 (1:50; MIB-1; DakoCytomation, Glostrup, Denmark) were used. Briefly, paraffin-embedded sections were dewaxed with xylene and gradually hydrated. Antigen retrieval was performed by autoclaving in 10 mM citrate buffer (pH 6.0) for 10 min after endogenous peroxidase activity had been blocked by immersing the sections in 0.3% hydrogen peroxide in methanol for 30 min. The sections were then reacted with each primary antibody overnight at 4°C, prior to being treated with secondary antibody and biotin-streptavidin complex (Nichirei Corporation, Tokyo, Japan) for 30 min each at 37°C. The resultant immunoreactions were visualized with diaminobenzidine (DakoCytomation, Glostrup, Denmark) and the sections were counterstained with hematoxylin (Wako Pure Chemical, Osaka, Japan).

### Evaluation of immunohistochemical observations

To evaluate PITX1 and Ki-67 expression, images of positive tumor cell nuclei were captured using a charge-coupled device camera (Nikon, Tokyo, Japan) in the most strongly labeled region. Subsequently, the number of positive cells was counted in high-magnification fields using the FLVFS Image Filing Software (Flovel, Co., Ltd., Tachikawa, Japan) and the percentage of positive cells was determined for each antibody. The numbers of normal squamous, dysplastic and OSCC cells were counted among >1,000 cells and the percentage of positively stained cells was designated as the labeling index (LI; %).

### Statistical analysis

The correlations between the PITX1 or Ki-67 LIs and malignant transformation were calculated using Pearson’s correlation test. Student’s t-test or Mann-Whitney U test were used for comparisons between two categorical variables. P<0.05 was considered to indicate a statistically significant difference.

## Results

### PITX1 expression levels in the normal oral mucosa, oral epithelial dysplasia and OSCC

**Figure 1:**
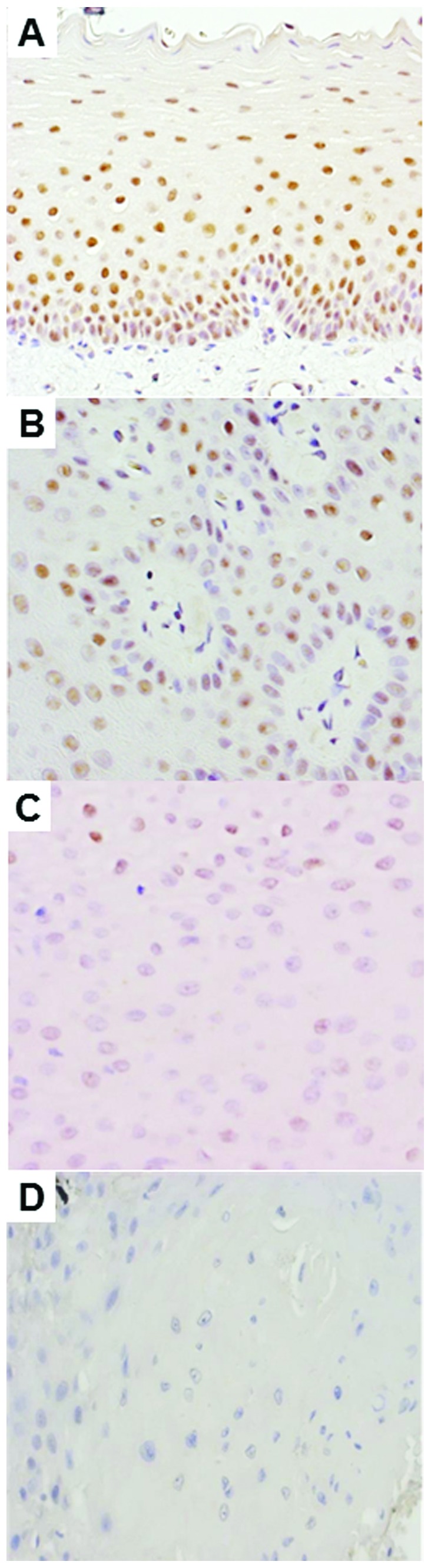
Immunohistochemical examination. Immunohistochemical staining of paired-like homeodomain 1 in the (A) normal oral mucosa, (B) oral epithelial dysplasia without malignant transformation, (C) oral epithelial dysplasia with malignant transformation and (D) oral squamous cell carcinoma (magnification, ×400).

**Figure 2:**
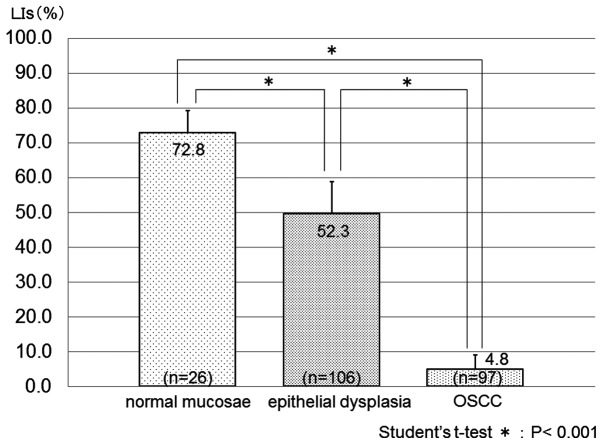
PITX1 expression levels in the normal oral mucosa, oral epithelial dysplasia and OSCC. The LIs of PITX1 were significantly decreased in oral epithelial dysplasia compared with the normal oral mucosa (P<0.001), and were further decreased in OSCC (P<0.001). PITX1, paired-like homeodomain 1; OSCC, oral squamous cell carcinoma; LIs, labeling indices.

Immunohistochemical analysis showed that PITX1-positive cells were distributed in the basal cell layer of the normal oral mucosa (Fig. 1A) and in numerous oral epithelial dysplasia samples (Fig. 1B). By contrast, only weak nuclear staining was detected in the OSCC (Fig. 1D) and sections of oral epithelial dysplasia samples (Fig. 1C). The PITX1 LIs of the normal oral mucosa, oral epithelial dysplasia and OSCC specimens are summarized in Fig. 2. The PITX1 LI was 72.8±6.5, 52.3±9.24 and 4.8±4.25 [mean ± standard deviation (SD)] in the normal oral mucosa, oral epithelial dysplasia and OSCC samples, respectively. The mean PITX1 LI of the oral epithelial dysplasia samples was significantly lower than that of the normal oral mucosa specimens, but significantly higher than that of the OSCC samples (P<0.001).

### Downregulation of PITX1 expression in oral epithelial dysplasia is a predictive marker of malignant transformation in OSCC

**Figure 3:**
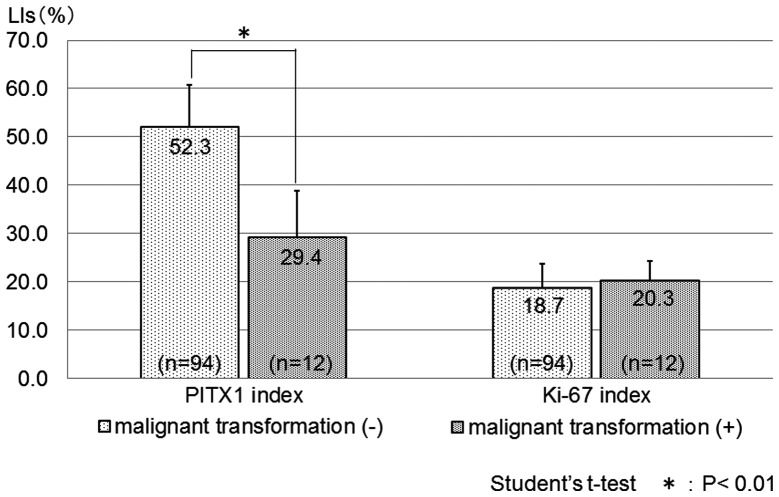
Correlation between the LIs of PITX1 and Ki-67 and malignant transformation in oral leukoplakia. The LIs of PITX1, but not Ki-67, were significantly lower in the malignant transformation-positive cases of oral epithelial dysplasia than in the malignant transformation-negative oral epithelial dysplasia cases (P<0.01). PITX1, paired-like homeodomain 1; LIs, labeling indices.

The malignant transformation of oral epithelial dysplasia into OSCC occurred in 12 (11.3%) of the 106 patients with oral epithelial dysplasia. Immunohistochemical analysis showed that the malignant transformation-negative oral epithelial dysplasia cases were detected for PITX1 nuclear staining (Fig. 1B). By contrast, only weak nuclear staining was detected in the malignant transformation-positive oral epithelial dysplasia cases (Fig. 1C). The PITX1 LI was 52.3±8.40 (mean ± SD) in the cases of oral epithelial dysplasia that did not undergo malignant transformation (P<0.01) and 29.4±9.40 in those that did (Fig. 3), and the difference was significant (P<0.01). In addition, low PITX1 expression was found to significantly correlate with malignant transformation.

By contrast, the Ki-67 LIs were 18.7±5.07 (mean ± SD) in the malignant transformation-negative oral epithelial dysplasia cases (P<0.01) and 20.3±3.99 in the malignant transformation-positive cases (Fig. 3). The difference between the two groups was not significant.

**Figure 4:**
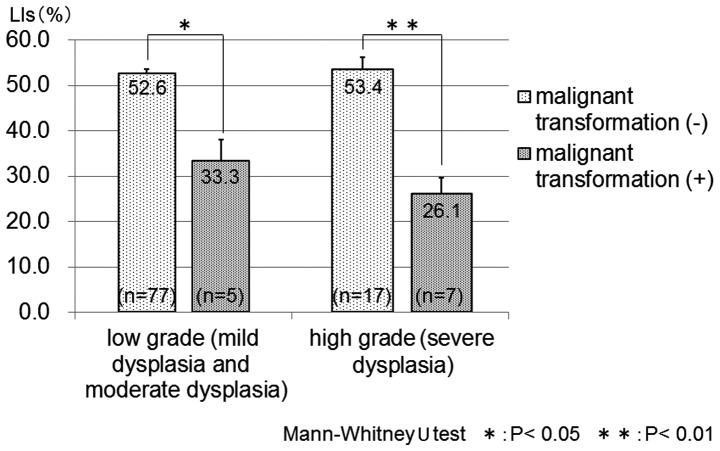
PITX1 expression levels in different histological grades of oral epithelial dysplasia. The mean LI of PITX1 in oral epithelial dysplasia with malignant transformation was significantly decreased, independent of the histological grade of the dysplasia. PITX1, paired-like homeodomain 1; LI, labeling index.

Next, the oral dysplasia cases were separated into low-grade (mild or moderate dysplasia) and high-grade (severe dysplasia) groups. In the low-grade group, the PITX1 LI was 52.6±0.94 [median ± standard error (SE)] for malignant transformation-negative oral epithelial dysplasia and 33.3±4.85 for malignant transformation-positive oral epithelial dysplasia. In the high-grade group, the PITX1 LI was 53.4±2.75 (median ± SE) for malignant transformation-negative oral epithelial dysplasia and 26.1±3.53 for malignant transformation-positive oral epithelial dysplasia. The median PITX1 LI was significantly decreased in malignant transformation-positive oral epithelial dysplasia, regardless of the histological grade of the dysplasia (Fig. 4).

## Discussion

The activation of oncogenes, inactivation of tumor suppressor genes and increased cellular proliferation are key events in the multi-step carcinogenesis process (27). The presence of oral epithelial dysplasia is generally accepted as one of the most important predictors of malignant development in premalignant oral lesions (1,4). In addition, the presence of certain genetic abnormalities in carcinoma-carrying patients may be an important prognostic indicator of patient survival. Such indicators may be useful in the clinical setting for selecting the initial treatment or developing tumor-specific therapies. Accordingly, a number of previous molecular studies have attempted to detect prognostic indicators for oral cancer. Several proteins, including Mcm2 (27,28), p53 (29), Bcl-2 family (30), ΔNp63 (31) and Ki-67 (29,32–34), have been identified as prognostic markers in oral epithelial dysplasia. In particular, a number of previous studies have examined Ki-67 expression in the transformation of oral epithelial dysplasia. The Ki-67 antigen was first reported by Gerdes *et al*, which is a nuclear protein associated with cellular proliferation (35). Ki-67 has since become one of the biomarkers frequently used as a prognostic indicator and proliferation marker (36). Ki-67 is a nuclear non-histone protein that is maximally expressed in cells in the G2 and M phases of the cell cycle, but is absent from resting cells. Therefore, Ki-67 may be employed to evaluate the percentage of proliferating cells in normal tissues, as well as premalignant and malignant lesions (34). Previous studies have suggested a strong correlation between Ki-67 expression and the degree of dysplasia, indicating that Ki-67 may be involved in proliferative events as well as neoplastic transformation (33). However, in the present study, the Ki-67 LI did not differ between the malignant transformation-positive and -negative cases of oral epithelial dysplasia, indicating that the Ki-67 LI is not useful as a prognostic marker in oral epithelial dysplasia. By contrast, it was found that PITX1 downregulation was significantly more common in malignant transformation-positive oral epithelial dysplasia cases than in malignant transformation-negative cases, independent of the histological grade of dysplasia.

The reduced PITX1 expression observed in the malignant cases suggested that PITX1 may have a tumor-suppressing function. This encouraged the current study to examine its potential role in the carcinogenesis of oral epithelium. Previously, Kolfschoten *et al* reported that PITX1 suppresses tumorigenicity by downregulating the Ras pathway through RASAL1, a member of the Ras-GTPase activating protein family of genes (GTP-activating negative regulators of Ras expression) (37). Activation of the Ras/mitogen-activated protein kinase (MAPK) pathway contributes to the tumorigenesis and progression of OSCC and inhibition of the pathway was found to suppress the proliferation of human OSCC (15). Therefore, it has been suggested that the loss of PITX1 expression results in the activation of the Ras/MAPK pathway via the downregulation of RASAL1, leading to the hyperproliferation of oral epithelia.

The present study was the first to report a correlation between PITX1 and oral epithelial dysplasia. The downregulation of PITX1 in oral epithelial cells may be involved in the carcinogenesis of OSCC. Thus, PITX1 is considered to be a candidate tumor suppressor gene. In addition, PITX1 may serve as a novel biomarker for predicting prognosis in oral epithelial dysplasia. Further studies must examine the mechanisms that modulated PITX1 expression during the progression of OSCC.
